# Memoirs on a Few Fundamental Points of Dental Medicine, Considered in Its Application to Hygiene and Therapeutics

**Published:** 1854-01

**Authors:** A. F. Talma, C. A. Du Bouchet

**Affiliations:** Dentist to the King of Belgium, &c.


### ARTICLE XXI.

*Memoirs on a few Fundamental Points of Dental Medicine, considered in its Application to Hygiene and Therapeutics.* By A. F. Talma, M. D., Dentist to the King of Belgium, &c. Translated by C. A. Du Bouchet, M. D., D. D. S. First Series. Brussels, 1852.

[Continued from October Number.]

The diseases of milk teeth frequently become the cause of accidents or complications, which hinder and render laborious the second dental eruption. The caries of the incisors and canines is generally of little importance, and may be neglected without inconvenience. Such is not the case with the molars. Among children, these teeth frequently decay several years before the natural epoch for their being shed. At the commencement of the caries, when the alteration of the tissues is still very limited, and when pain has not yet been experienced, it is proper, if the disposition of parts allows it, to fill them with much care. This operation has the twofold advantage—first, to preserve for a longer time organs useful for mastication, and the regular development of the dental arch; second, to save the patients those frequent paroxysms of pain, which must have a bearing upon the health. Beneath the metal obturating the caries, and sheltered from the contact of air and articles of food, the morbid labor is checked, if not entirely stopped, and the dental pulp is gradually destroyed in an insensible manner.

When the caries is already deeply seated, and when the cavity it has dug out is painful, or merely irritated by the contact of an instrument, a favorable opportunity to fill it no longer exists. At this period, the operation of filling would almost infallibly cause the exasperation of the morbid condition, constant abscesses, or other accidents, which might render a premature extraction necessary. It is essential to avoid such a result or termination.

As a sequel to such affections, in lymphatic or cachectic children, it is not rare to notice the appearance of denudations of the alveoli, and partial necrosis of the alveolar ridges, or even complete necrosis to a lesser or greater extent. The inflammation of the teeth is gradually extended from the gum to the periosteum, thence to the bony tissue, and causes denudations, and sometimes ill-looking ulcers, recalling certain forms of cancer. The permanent teeth, making their appearance under such circumstances, often remain loose or require to be extracted soon after their eruption. We should add that the affections we have spoken of are the more ordinarily local, and do not appear to have any bearing upon the system.

A strengthening diet, bitter tonics, detersive applications, a great cleanliness of the mouth, and when the pain becomes intolerable, the extraction of the carious teeth, are the means to be employed to obtain a restoration to health of the diseased parts.

I must not omit, in closing this paper, a general remark of the highest importance; I have constantly noticed, that in cases similar to those just now described, hygiene, either as a prophylactic or curative means, is the most useful and rational remedy. Not only the constitution of the teeth, but also their easy eruption, are dependent upon the conditions of health and vigor of the young subjects. Delicate, irritable children, born and raised in large cities, suffer most by their second dentition, as well as by their first. Hence the necessity of creating or establishing for them, as far as possible, a healthy and vigorous constitution, by means of continual exercise in the open air, frequent baths, healthy food, and a well-regulated physical education.

III.—*Anomalies.*—I shall but briefly allude to the anomalies of which the teeth have given examples, respecting the various periods of their appearance, their number, their continuation in the mouth, or their shedding. Most of these deviations from the general, natural law, depend from individual organic conditions, so deeply hidden that, most commonly, we can only oppose their results, but are unable to prognosticate them and oppose prophylactic means.

Like that of deciduous teeth, the eruption of the permanent teeth is precocious or slow. A great number of celebrated men are cited by biographers as having been born with incisors and even molars. Louis the Fourteenth was like the first, and Mirabeau like the latter. Among the ancients this phenomenon indicated an uncommon vigor, and, although comprising many exceptions, this belief is, however, in conformity with the most general observations. As regards the second dentition, it is not rare either to see permanent teeth cut in advance of the usual time, even at times when the corresponding deciduous teeth are yet perfectly firm, or to observe their tardiness to appear long after the premature loss of the deciduous ones, or even their entire deficiency. M. Duval relates the extraordinary case of a magistrate of Frederichstadt, who never cut his permanent molars, and was entirely deprived of incisors and canines.

I have seen an upper canine cut at the age of twenty-five, two inferior bicuspids at thirty, and wisdom teeth, in the case of a lady, at sixty. There is hardly any dentist who has not witnessed anomalies of this kind, of which it would be difficult to give a satisfactory explanation. For it cannot well be understood on what account the growth of certain teeth, arrested during many years, should resume, at a given point, its activity, increase the length of their roots, and compel the part clothed with enamel to burst forth and overcome the very considerable resistance of the tissues completely covering them.

The delay noticed in the eruption of deciduous teeth, sometimes appears to be in consequence of the weakness of the constitution, and the debility of organic movements in certain lymphatic and delicate subjects. M. Duval has related a case precisely in point.

Alphonse Leroy has also stated, in his "Treatise of Maternal Medicine," that the dentition is delayed, if the child be puny and the issue of debilitated parents, or insufficiently fed.

The want of permanent teeth has been noticed, not merely partial but total. It may depend either from the primitive absence of dental germs in the fœtal organization, or from diseases severe enough to destroy those germs, during the early periods of life. M. Oudet has found, in his anatomical researches on the fœtus, the dental germs inflamed and suppurating, and as this skillful observer remarks: "There is not much question that, had those subjects lived, they would have been deprived of the teeth whose rudiments were thus destroyed, or in process of destruction." These facts recall what I have already mentioned in regard to the influence exercised by pathological affections, more or less grave, on the teeth undergoing the process of formation. In such cases, where that influence is exerted to a high degree, the teeth, instead of merely undergoing alterations in their tissues, may be completely destroyed. M. Maingault has related the case of a lad of the age of eighteen, whose permanent teeth had not yet appeared. M. Murat saw an adult, whose deciduous teeth, almost entirely worn off, still persisted.

"Nature," says with much reason, M. Duval, "sometimes preserves deciduous teeth to fill the place of permanent ones. The roots of such teeth are not absorbed like those that are shed, and they remain, *in situ*, with some mobility. Usually shorter, more yellow and worn than the others, they remain in the mouth until the age of forty and upward. This phenomenon is observed particularly in regard to the deciduous molars; it is very important to bear it in mind, so as not to uselessly extract them."

The cases in which deciduous teeth are either not shed, or are so at a late period, are exceptions, but they are numerous enough to deserve notice.

The exaggerated number of teeth is most ordinarily owing to the abnormal persistency of a few deciduous teeth, anteriorly or posteriorly to which the permanent teeth were to arrange themselves. These cases, not uncommon, merit all the attention of the operator. The deciduous tooth should be removed in every case rather than sacrifice a permament one; the permanent tooth will naturally, or with the assistance of art, resume its usurped place. But it is not always perfectly easy to discriminate which is which, and I have seen in similar cases, very deplorable mistakes made. Although paying due attention to the account of the case, as related by the patient or friends, the dentist should make a very thorough examination of the characteristics of the teeth regarding which any doubt may exist. Permanent teeth, relatively to deciduous or milk teeth, are more voluminous, of a less milky white, of a more solid appearance; not having undergone wear by usage, their anfractuosities are sharper. These peculiarities, which habit teaches us how to distinguish, must be noted with care, in order to avoid grave errors, unluckily too often committed.

Real supernumerary teeth are rare. Besides, the fabulous cases of double rows of teeth related by the ancient writers, more modern ones have been mentioned, which we cannot reject. * * * * * *

When supernumerary teeth produce neither deformity nor inconvenience of any kind, we should not interfere with them. But when they do, which usually happens in regard to canines, and far more so in the case of molars, art must be put in requisition. According to the situation and direction which they assume, supernumerary teeth frequently hinder the motions of the tongue and cheeks; they irritate those parts, ulcerate them sometimes, and become the incessant cause of suffering, or, at least, great inconvenience. In such cases the indication is obvious; they must be extracted, although the operation may not be exempt from difficulties.

Among the anomalies sometimes presented by teeth of the second dentition, I could not omit the union, or rather confusion, of several teeth into one. The cases of dental rows composed of one single piece, related by the ancients, must be rejected by sober science. But it is not the same with the isolated soldering of a few teeth in consequence of the abnormal approximation and growing together of their still vesicular germs. M. Oudet, and several other dentists, have observed cases of this kind. The most remarkable I have noticed, was a central and a lateral upper left incisors, inserted into one large tooth, with hardly any indentation at the line of junction. M. M. Desirabode thinks that the canine and lateral incisors are the teeth most liable to unite. These cases are curious, but offer no practical indication.

The annals of science have preserved the history of a few aged persons in whom nature has retrieved, by the means of a sort of third dentition, the loss of adult teeth worn out by age, or spontaneously expelled by the atrophy of the gums. These supplementary productions, extremely rare, are almost always irregular, partial, and more or less deformed abortions. Their development is seldom a benefit, and they furnish no useful assistance in the performance of functions. Far from that, scattered upon a barren alveolar ridge, without support from neighboring teeth, having no antagonists in the opposite jaw, loosely set in their position, they usually rather hinder mastication than facilitate it, irritate the gums, and even sometimes cause such inconvenience that their extraction becomes imperative.

We do not see these anomalies only among the aged subjects; sometimes, also, children and adults display these freaks of nature. Among the most curious examples of these multifarious dentitions, I will quote that, which a respectable practitioner has related in the *Bulletin de Therapeutique*, for September,

1837. This fact exhibits at the same time, the exceptional fecundity of nature and the fragility of its too often repeated products. This case is that of a child, thirteen years old, whom the doctor has seen and known from his birth. This child, says Dr. Licon, a physician of Donzy, accomplished his second dentition at the age of nine. Soon after, several teeth became loose, and displayed new teeth. The twenty-eight teeth were thus shed and renewed in a short time. Between the age of ten and eleven, the same phenomenon occurred a second time. From eleven to twelve, same shedding and removal of all the teeth. Finally, at the time when that child reached his thirteenth year, a sixth dentition was progressing; the first large molar being shed, being expelled by a similar one, quite visible. The teeth which nature thus renewed so promptly had no roots; they seemed to have been entirely absorbed; destroyed. Their shedding and the consecutive eruption took place in the usual order. The child was well, and experienced no annoyance. The gums, over-taxed by this continuous labor, were rather red and swollen. The teeth were small, white, and regularly arranged.

We cannot doubt but facts of this kind arise only from an abnormal prodigality in the number of germs intended for successive development. Lemaire found at the extremity of the root of a superior canine, which he extracted for a girl of sixteen, three other much smaller teeth, which had grown with the former. M. Oudet, having removed a voluminous tumor, implanted at the right side of the lower jaw, found it to contain at least twenty-five teeth, distinct from each other in form, position and direction. They were united together either by direct contact, or by a solid reddish-yellow substance. It is evident that in this case, had circumstances permitted, there would have been, as in Mr. Licon's case, a series of more or less numerous dental eruptions, probably quite as imperfect.

However, cases of this description are far from being all properly authenticated. The fondness of men for the marvelous must have allowance made; we can appreciate these cases only from accounts relating to distant periods, and it is not strange that with the help of the imagination, the mere approximation of two neighboring teeth may have been taken for a new eruption. The dentist would commit a very serious error if, supposing such exceptional facts to be positively exact, he lightly and without absolute necessity sacrificed, even in the case of young subjects, permanent teeth, in the hope of seeing them replaced by new ones. Nature does not thus keep in reserve, at our wish, supplementary follicles, in order to retrieve eventual losses. In this respect, there is no more prospect of replacement for adolescence than for old age.

IV.—*Arrangement of the Teeth.*—Having prepared, as far as practicable, by hygienic cares, a good constitution and the easy eruption of the permanent teeth, overcoming, according to the nature of the case, the accidents which may follow from the eruption of these organs, it is important to follow and observe with care the progress of the second dentition, so as to prevent or correct in the outset the irregularities which may occur.

Owing to an admirable mechanism, nature, although maintaining during first infancy the anterior portion of the dental arches in the same dimensions, has not, however, debarred the body of the jaws from the general laws of organic development. On one part, the deciduous teeth have been enabled to preserve, until being shed, their primitive relations, and on the other, the deeper soil—if I may thus express myself—inclosing, and from which the permanent teeth are to issue forth, has increased in proportion to the size required by the latter. This enlargement, which continues until the complete formation of the bony structure, proceeds in every direction, and carries the maxillary angles backwards as well as the symphysis forward.

The special labor for the growth of the germs and crowns of the permanent teeth constitutes, for the jaws, and especially for the alveolar ridges, a local cause, active and powerful, for development. Larger than their predecessors, the permanent teeth make for themselves, by main force, thus to speak, the room necessary for them in the dental arch. Hence the advantage resulting, for their regular arrangement, from their erupting successively and in proportion to the motion of extension operated in the whole system.

I propose to explain, in a subsequent memoir, with all the requisite details, the history of the multifarious causes, more or less deeply situated, occasioning the deformities of the mouth, which the dentist may be called upon to remedy. I could, at this point, indicate those causes, but in an incomplete manner, making myself liable to useless repetitions. I shall, then, now confine myself only to the direction of second dentition, in the most ordinary cases, and beyond, the normal organic dispositions which may interfere with it.—*Dental News Letter.*

[To be continued.]

